# Current News

**Published:** 1894-03

**Authors:** 


### Current News.

### THE GENERAL MEDICAL COUNCIL OF GREAT BRITAIN.

"The Committee considered applications from the following persons, who hold American dental qualifications recognized by the Council prior to May 29, 1893, and who urge that they should be allowed to be registered on the ground of having commenced their course of study for their diplomas in several cases long previously, in the belief that they would constitute registrable qualifications, and received no sufficient notice to the contrary.

"George William Field, D.M.D., University of Harvard; Edward M. Quinby, D.M.D., University of Harvard; G. Rufus Gray, D.M.D., University of Harvard; L. N. Seymour, D.D.S., University of Michigan; B. C. Hinkley, D.D.S., University of Michigan; E. V. Hinkley, D.D.S., University of Michigan; E. G. Snodgrass, D.D.S., University of Michigan.

"*Resolved*, That these gentlemen be informed that they cannot be admitted to registration unless they can prove that they had passed through a curriculum equivalent to that demanded by the Medical Council from the licensing bodies of the United Kingdom."

[The remarks upon this by the editor of *The Journal of the British Dental Association* indicates very clearly the temper of the dental profession in England.—Ed.]

"The refusal of the Council to accede to the request of seven gentlemen who had obtained American dental qualifications recognized by the Council prior to May, 1893, and who urged that they should be allowed to be registered, on the ground of having commenced their courses of study for these diplomas previously to that date, in the belief that they would prove registrable qualifications 'unless they can prove that they had passed through a curriculum equivalent to that demanded by the Medical Council from the licensing bodies of the United Kingdom,' shows that the Council is in earnest with regard to its decision of May last upon this question, and is not prepared to go back upon it. *To us the applications prove how urgent was the necessity for a change in the administration of this portion of the Dentists' Act.*" (Italics ours.)

### NEW JERSEY STATE DENTAL SOCIETY.

*Committees for 1894.*

*Accommodations.*—Dr. Charles A. Meeker, Dr. C. W. F. Holbrook.

*Programmes.*—Dr. Charles A. Meeker.

*Clinics.*—Dr. R. M. Sanger, Chairman; Dr. O. Adelberg, Dr. A. R. Eaton.

*Exhibits.*—Dr. C. W. F. Holbrook, Chairman; Dr. Harvey Iredell, Dr. B. F. Luckey.

*Essays.*—Dr. S. C. G. Watkins, Chairman; Dr. C. S. Stockton, Dr. J. Allen Osmun.

*Clinical Conference.*—Dr. Harvey Iredell, Chairman; Dr. Wm. E. Truex, Dr. J. W. Curtis, Dr. Thomas Moore, Dr. I. M. Vandewater.

*Dental Literature.*—Dr. A. R. Eaton, Chairman; Dr. George E. Adams, Dr. W. Woolsey.

*Mechanical Appliances.*—Dr. Wm. P. Richards, Chairman; Dr. Henry A. Hull, Dr. F. W. Kitchell.

*Materia Medica.*—Dr. Charles A. Meeker, Chairman; Dr. G. Carleton Brown, Dr. G. E. Adams.

*Prosthetic Dentistry.*—Dr. F. C. Barlow, Chairman; Dr. H. B. Van Dorn, Dr. P. J. Wilson, Dr. J. L. Crater.

*Transportation.*—Dr. George C. Brown.

*Press.*—Dr. F. C. Barlow.

*Inspection in the Public Schools, etc.*—Dr. Wm. L. Fish, Chairman; Dr. W. E. Linstead, Dr. F. L. Hindle, Dr. A. A. Schubert.

*Legislative Committee.*—Dr. Charles A. Meeker; Dr. F. C. Barlow; Dr. James C. Clarke; Dr. George C. Brown; Dr. R. H. Sheppard; Dr. A. R. Eaton. (Chairman to be elected).

*Entertainment Committee.*—Dr. B. F. Luckey, Chairman; Dr. Wm. L. Fish, Dr. F. E. Riley, Dr. O. Adelberg, Dr. Wm. E. Truex, Dr. George E. Adams, Dr. Wm. E. Linstead.

The chairmen of the respective committees will at once communicate with the several members of their committees and arrange for the work to be performed. The Secretary will make a call for results May 26. Meetings of the several committees can be called for the afternoons of February 19 and April 16, from 2 to 6 o'clock. The use of rooms on the third floor of S. & J. Davis's, No. 943 Broad Street, has been tendered the committees.

### OHIO STATE DENTAL SOCIETY.

At the last annual meeting of the Ohio State Dental Society, held December, 1893, the following officers were elected for the ensuing year, 1894:

President, Chas. Welch, Wilmington; First Vice-President, W. H. Todd, Columbus; Second Vice-President, Henry Barnes, Cleveland; Secretary, L. P. Bethel, Kent; Assistant Secretary, L. E. Custer, Dayton; Treasurer, C. I. Keely, Hamilton.

L. P. Bethel,
*Secretary.*

---

### WOMAN'S DENTAL ASSOCIATION OF THE UNITED STATES.

The regular monthly meeting of the Woman's Dental Association was held February 3, 1894, at 1308 Walnut Street, Philadelphia, the President, Dr. Mary H. Stilwell, in the chair.

In the absence of the essayist, Dr. Alice I. Ireland, her paper was read by the Corresponding Secretary, Dr. Annie T. Focht; subject of paper, "Food: Its Relation to the Teeth."

Next meeting will be held March 3, 1894, at 1300 Arch Street, Philadelphia. It will be the yearly business meeting.

Eliza Yerkes,
*Recording Secretary.*

4004 Chestnut Street, Philadelphia.

---

### ST. LOUIS DENTAL SOCIETY.

The St. Louis Dental Society will hold its annual clinics on March 20, 21, and 22, 1894.

Reduced railroad rates. A cordial invitation is extended to all members of the profession.

For further particulars, address,

John G. Harper, *Corresponding Secretary,*
803 Pine Street, St Louis, Mo.

### INTERNATIONAL MEDICAL CONGRESS.

The Eleventh International Medical Congress will be held at Rome, Italy, March 29 to April 5, 1894.

The sections number nineteen. Odontology being number thirteen on the list.

---

### ILLINOIS STATE DENTAL SOCIETY.

The Thirtieth Annual Meeting of the Illinois State Dental Society will be held in the Senate Chamber, Springfield, Ill., May 8, 9, 10, and 11, 1894. An interesting programme is in the course of preparation. Practitioners of Illinois and of adjoining States are cordially invited to attend.

Louis Ottofy,
*Secretary.*

---

### RECENT PATENTS.

A list of recent patents, reported specially for the International Dental Journal:

509,901.—Extensible Dental-Engine Bracket. Horace Hobbs, Milwaukee, Wis., assignor of one-half to Augustus W. Friese, same place. Filed August 22, 1892.

510,048.—Electrical Apparatus for Operating Dental Implements. Oscar H. Pieper, San José, Cal. Filed November 25, 1892.

510,325.—Device for Measuring Tooth-Roots. Asher I. F. Buxbaum, Cincinnati, Ohio. Filed February 17, 1893.

510,484.—Dental Engine. Leroy S. Pfouts, Canton, Ohio. Filed January 13, 1893.

510,856.—Brake for Dental Engines. Peter V. Guerry, Philadelphia, Pa. Filed June 30, 1893.

510,963.—Dentists' Device for Saving Gold Fillings. Alonzo J. Douds and Frank F. Douds, Canton, Ohio. Filed January 31, 1893.

511,029.—Dental Hand-Piece. Christopher M. Spencer, Windsor, Conn. Filed September 27, 1893.

511,067.—Dental Forceps. Harry Walter, Philadelphia, Pa., assignor to George W. Teufel, same place. Filed March 23, 1893.

511,619.—Teeth-Separator. James W. Ivory, Philadelphia, Pa. Filed May 11, 1891.

